# Melatonin intervention to prevent delirium in the intensive care units: a systematic review and meta-analysis of randomized controlled trials

**DOI:** 10.3389/fendo.2023.1191830

**Published:** 2023-07-26

**Authors:** Yushan Duan, Yuan Yang, Weihua Zhu, Linjun Wan, Gang Wang, Jinxi Yue, Qi Bao, Jianlin Shao, Xiaohong Wan

**Affiliations:** ^1^ Department of Critical Care Medicine, The Second Affiliated Hospital, Kunming Medical University, Kunming, China; ^2^ Department of Anesthesiology, The First Affiliated Hospital, Kunming Medical University, Kunming, China

**Keywords:** melatonin, delirium, intensive care, meta-analysis, randomized controlled trials

## Abstract

**Objective:**

To determine the preventive effect of melatonin on delirium in the intensive care units.

**Methods:**

We conducted a systematic search of the PubMed, Cochrane Library, Science, Embase, and CNKI databases, with retrieval dates ranging from the databases’ inception to September 2022. Controlled trials on melatonin and placebo for preventing delirium in the intensive care units were included. The meta-analysis was performed using Review Manager software (version 5.3) and Stata software (version 14.0).

**Results:**

Six studies involving 2374 patients were included in the meta-analysis. The results of the meta-analysis showed that melatonin did not reduce the incidence of delirium in ICU patients (odds ratio [OR]: 0.71; 95% confidence interval [CI]: 0.46 to 1.12; p = 0.14). There was a strong hetero-geneity between the selected studies (I^2^ = 74%). Subgroup analysis results showed that melatonin reduced the incidence of delirium in cardiovascular care unit (CCU) patients (OR: 0.52; 95% CI: 0.37 to 0.73; p=0.0001), but did not in general intensive care unit (GICU) patients (OR: 1.14; 95% CI: 0.86 to 1.50; p=0.35). In terms of the secondary outcomes, there were no significant differences in all-cause mortality (OR: 0.85; 95% CI: 0.66 to 1.09; p=0.20), length of ICU stay (mean difference [MD]: 0.33; 95% CI: -0.53 to 1.18; p=0.45), or length of hospital stay (MD: 0.51; 95% CI: -1.17 to 2.19; p=0.55).

**Conclusion:**

Melatonin reduced the incidence of delirium in CCU patients, but did not significantly reduce the incidence of delirium in GICU patients.

**Systematic Review Registration:**

https://www.crd.york.ac.uk/prospero/, identifier CRD42022367665.

## 1 Introduction

Delirium has been defined as an acute impairment of attention and overall cognitive function in hospitalized patients (1). In critically ill patients, delirium is associated with increased morbidity and mortality, and may also increase the length of stay in critically ill patients (2–7). Delirium is especially common in intensive care unit (ICU) patients, with such a wide range of occurrence reported in the literature (11%-80%) (6, 8–10). Importantly, most medications currently available clinically are not effective for delirium and there is a lack of evidence-based medicine in the treatment of this disorder.

Melatonin, a serotonin-derived hormone secreted by the pineal gland, is a powerful free radical scavenger and antioxidant with a broad spectrum (11) that easily crosses the blood-brain barrier in large, non-toxic doses (12). It plays a major role in sleep regulation and circadian rhythm management (13, 14). Melatonin has anti-inflammatory and immunomodulatory properties and may have neuroprotective effects in brain injury, encephalopathy and neurodegenerative diseases (15–19).

Melatonin supplementation may prevent delirium. To date, there were many clinical trials of melatonin or melatonin agonists to prevent delirium (20–29). Due to the heterogeneity of experimental methods and patients, results between clinical trials are conflicting and it is difficult to draw firm conclusions. In light of recently published large randomized clinical trials (30), we conducted a comprehensive systematic review and meta-analysis to summarize the available evidence and provide more reliable quantitative results as a basis for delirium prevention and clinical treatment in critically ill patients.

## 2 Materials and methods

### 2.1 Search strategy

We conducted a systematic search of the PubMed, Cochrane Library, Science, Embase, and CNKI databases, with retrieval dates ranging from the databases’ inception to September 2022. We used the following search terms: “Delirium”, “Subacute Delirium”, “Delirium, Subacute”, “Deliriums, Subacute”, “Subacute Deliriums”, “Delirium of Mixed Origin”, “Mixed Origin Delirium”, “Mixed Origin Deliriums”, “Melatonin”[Mesh] and “Melatonin”. Search strategies were adapted for the various search engines. Manual retrieval from the references of subject-related articles was performed to broaden the search. We did not restrict our search by region or language. Two re-viewers (Y.D. and Y.Y.) independently assessed each included study, and any discrepancies were resolved through consensus.

### 2.2 Inclusion/exclusion criteria

Following the PICOS principle, inclusion criteria: (1) adult ICU inpatients; (2) delirium diagnosis criteria determined by CAM or CAM-ICU; (3) intervention: intervention group received melatonin enteral administration; control group received placebo enteral administration; (4) no statistically significant pre-experimental differences between experimental and control groups; (5) full paper containing at least one outcome parameter: incidence of delirium (primary outcome) and secondary outcomes: all-cause mortality, length of ICU stay, and length of hospital stay; (6) study type was a randomized controlled trial, or cohort study. Exclusion Criteria: reviews, case reports, letters, low-quality researches and researches with no detailed data.

### 2.3 Data extraction

We extracted the following data from the studies in the meta-analysis: author, publication year, study design, basic information, incidence of delirium, all-cause mortality, length of ICU stay, and length of hospital stay. If a continuous variable was present, the mean value and standard deviation were calculated.

### 2.4 Quality assessment

**Figure 1:**
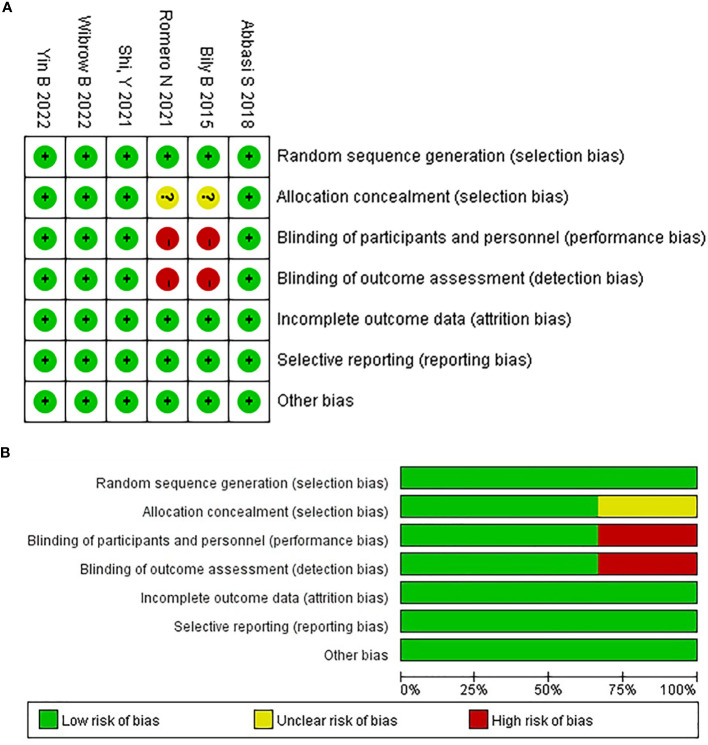
The risk of bias assessment for each trial using the risk of bias in Cochrane systematic reviews. **(A)** Risk of bias summary, **(B)** Risk of bias graph.

The risk of bias in Cochrane systematic reviews was used to evaluate the quality of the literature, in accordance with the guidelines for doing research in evidence-based medicine (31). Using six criteria randomization method, concealment of allocation scheme, blinding, completeness of outcome data, selective reporting of study results, and other sources of bias. The quality of the included studies was evaluated. Studies with fewer than five items were categorized as having a low risk of bias, those with three to four items as having a moderate risk of bias, and those with more than three items as having a high risk of bias. All six articles were of a high caliber, with four having a low risk of bias and the other two all having a moderate risk. In **Figure 1A**, the standard is “+”, and the non-compliance is “-”. **Figure 1B** shows the proportion of each item in the methodological assessment.

### 2.5 Statistical analysis

A meta-analysis of the included studies was performed using Review Manager software (version 5.3) and Stata software (version 14.0). For dichotomous variables, the odds ratio (OR) and 95% confidence interval (CI) were used as efficacy indicators for statistical analysis. Heterogeneity between studies was assessed using the Q-test and I^2^-test. If p< 0.05 or I^2^>50%, The data were combined using the random-effects model with the assumption that there was heterogeneity; however, if (p≥0.05 or I^2^<50%), data were integrated using a fixed-effects model with no consideration given to the existence of heterogeneity. P<0.05 indicated that differences were statistically significant. In order to examine potential publishing bias, a funnel plot was created.

### 2.6 Registration

The study was registered on PROSPERO (CRD42022367665).

## 3 Results

### 3.1 Study characteristics

**Figure 2:**
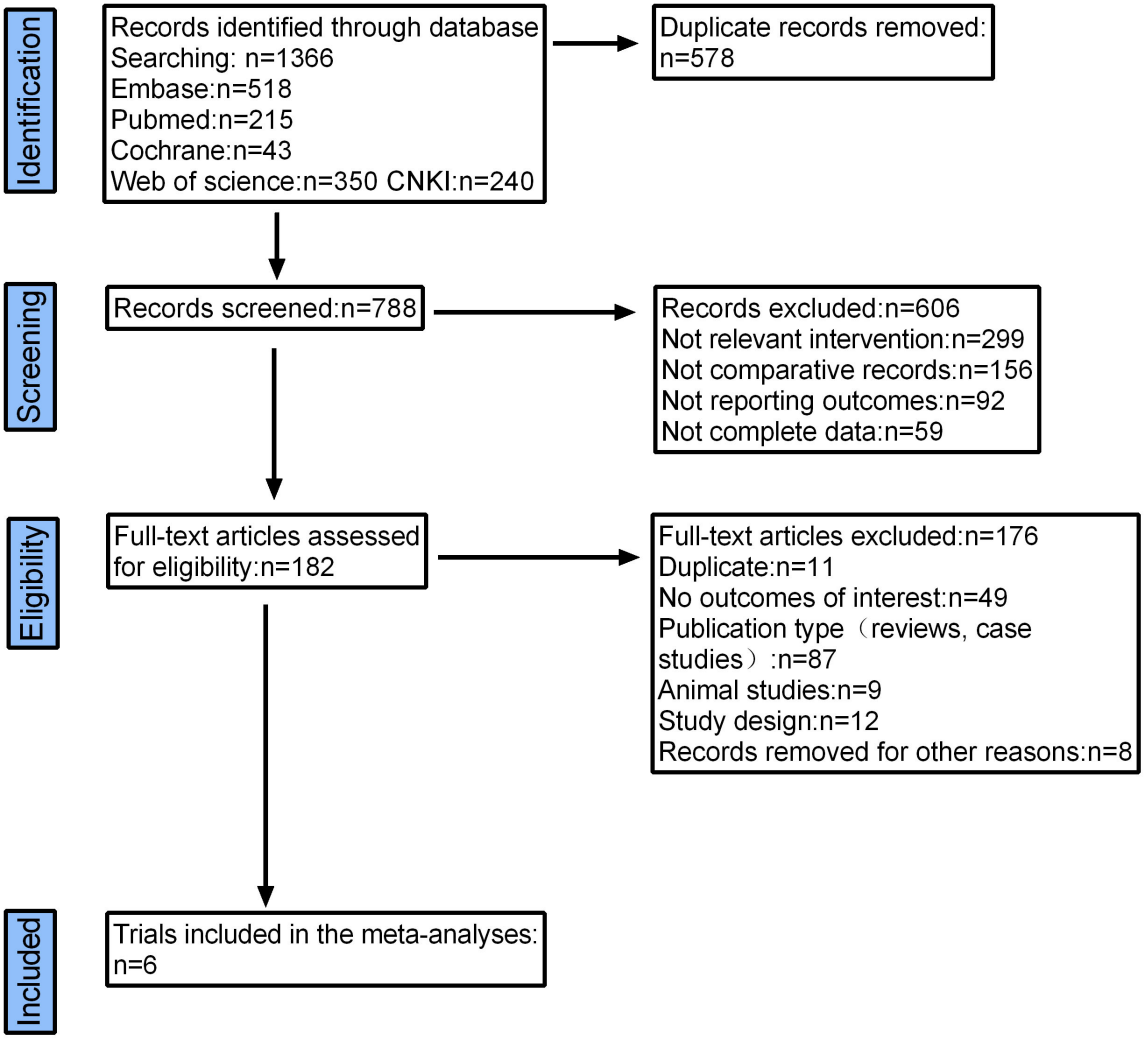
Flow diagram of studies identified, included and excluded.

A total of 1366 articles were initially retrieved. But 1184 articles were excluded because there were duplicates or irrelevant to our study. After reading the full text, another 176 articles were excluded. Finally, six studies with 2374 patients were included in our meta-analysis (26, 30, 32–35) (**Figure 2**). The characteristics of the included studies are shown in **Table 1**. The risk of bias in the Cochrane systematic reviews was used to evaluate the quality of the studies. All studies had a risk of bias, but most were low, and the average quality of each study was good. The results are shown in **Figure 1**.

**Table 1:** Baseline characteristics of include studies and methodological assessment.

Author and year	Study design	IIntervention	Dosage	Sample size (n)	Sex(male)	Age (years)	Delirium diagnosis tool
Wibrow B (2022)	RCT	Melatonin	4mg at 21h (for 14 continuous days)	419	247	61.9±15.1	CAM-ICU
		Placebo		422	280	61.9±15.2	
Yin B (2022)	RCT	Melatonin	3mg (for 7 continuous days)	248	152	69.1±7.5	CAM-ICU
		Placebo		249	143	68.5±7.1	
Romero N (2021)	Retrospective	Melatonin	at least 1 dose(median 3mg)	75	46	65.3±15.8	CAM-ICU
		Placebo		27	15	60.7±18.4	
Shi, Y (2021)	RCT	Melatonin	3mg (for 7 continuous days)	148	93	71.5±6.7	CAM
		Placebo		149	89	71.6±6.6	
Abbasi S (2018)	RCT	Melatonin	3mg at 21h (for 5 continuous days)	67	36	52.5 ± 18.4	CAM-ICU
		Placebo		70	42	49.9 ± 19.0	
Bily B (2015)	Prospective	Melatonin	5mg (for 4 continuous days)	250	179	64.3 ± 10.1	CAM-ICU
		Placebo		250	171	65.2 ± 10.3	

RCT, randomized controlled trials; X±Y, mean ± standard.

## 4 Incidence of delirium

**Figure 3:**
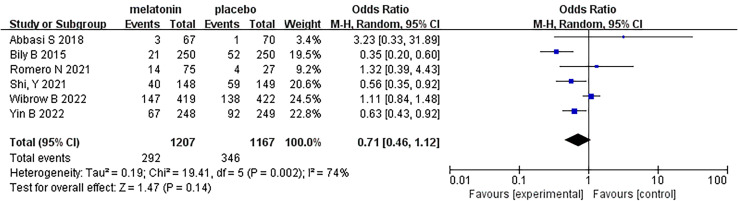
Forest plot and meta-analysis of incidence of delirium.

Six articles were analyzed, and a total of 2374 patients participated in the study, with 1207 patients receiving melatonin and 1167 patients receiving placebo. After the heterogeneity test, I^2^ = 74%>50%, and the P=0.002<0.1 of the Q test, suggested a strong heterogeneity between the selected studies, selecting random effects for meta-analysis, while continuing to investigate the reasons of the heterogeneity. Based on the data of this study, the source of heterogeneity was highly suspected to be inconsistent reasons for patients’ admission to the ICU, and subgroup analysis will be conducted according to the type of ICU. In terms of the type of ICU, patients were divided into two subgroups: general intensive care unit (GICU) group and cardiovascular care unit (CCU) group according to the reasons for their admission to the ICU. For the overall six articles, random effects were selected for me-ta-analysis and the results were shown in **Figure 3**. The results of the meta-analysis showed that melatonin did not reduce the incidence of delirium in ICU patients (Random-effects model: OR, 0.71; 95% CI, 0.46 to 1.12; p=0.14; I^2^ = 74%).

### 4.1 Sensitivity analysis

**Figure 4:**
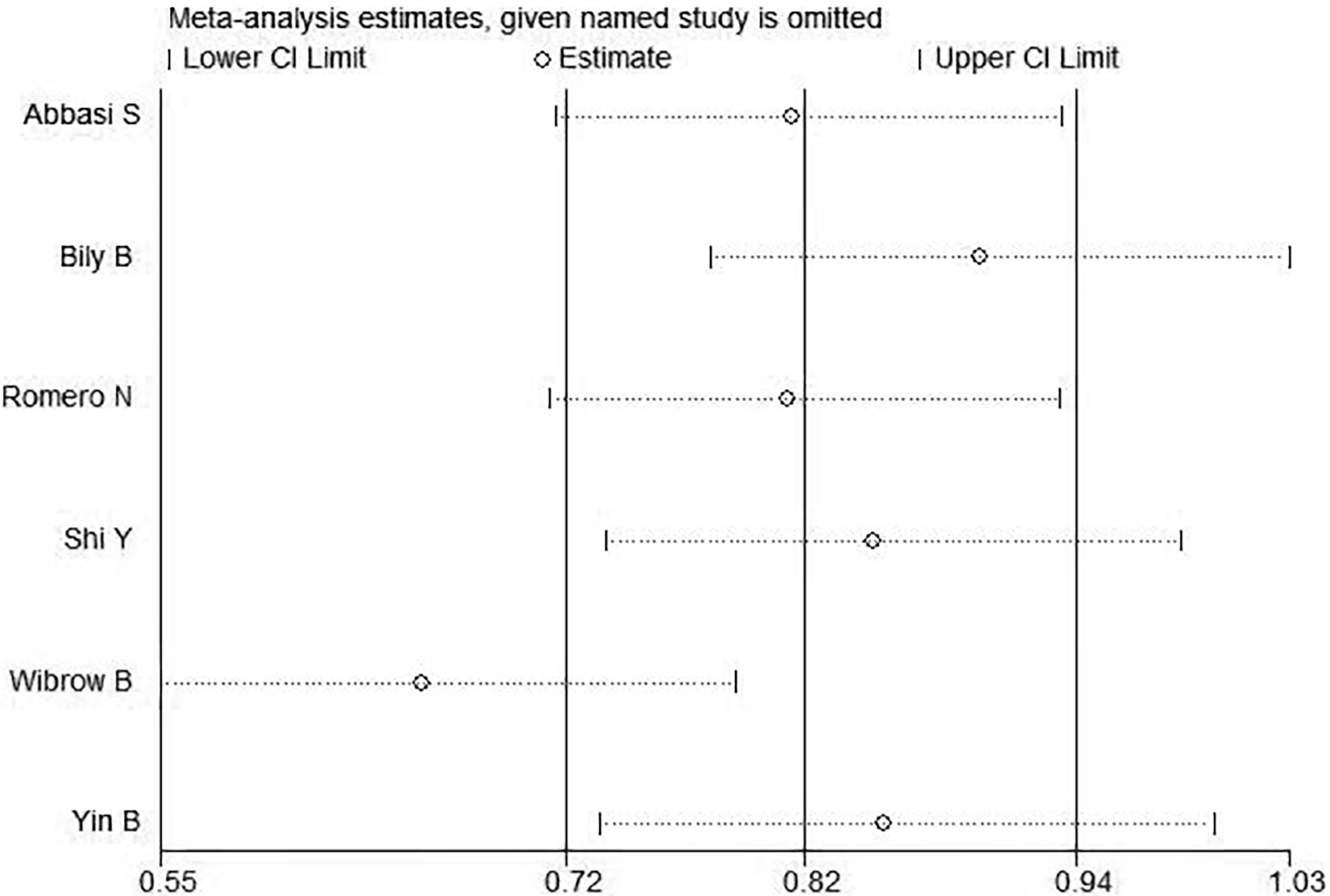
Sensitivity analysis of incidence of delirium.

The results of sensitivity analysis were shown in **Figure 4**. As could be clearly seen from the above figure that there were two distinct groups, the studies show different sensitive situations based on different ICU types. Therefore, it was highly suspected that the different types of ICUs cause heterogeneity.

### 4.2 Subgroup analysis

**Figure 5:**
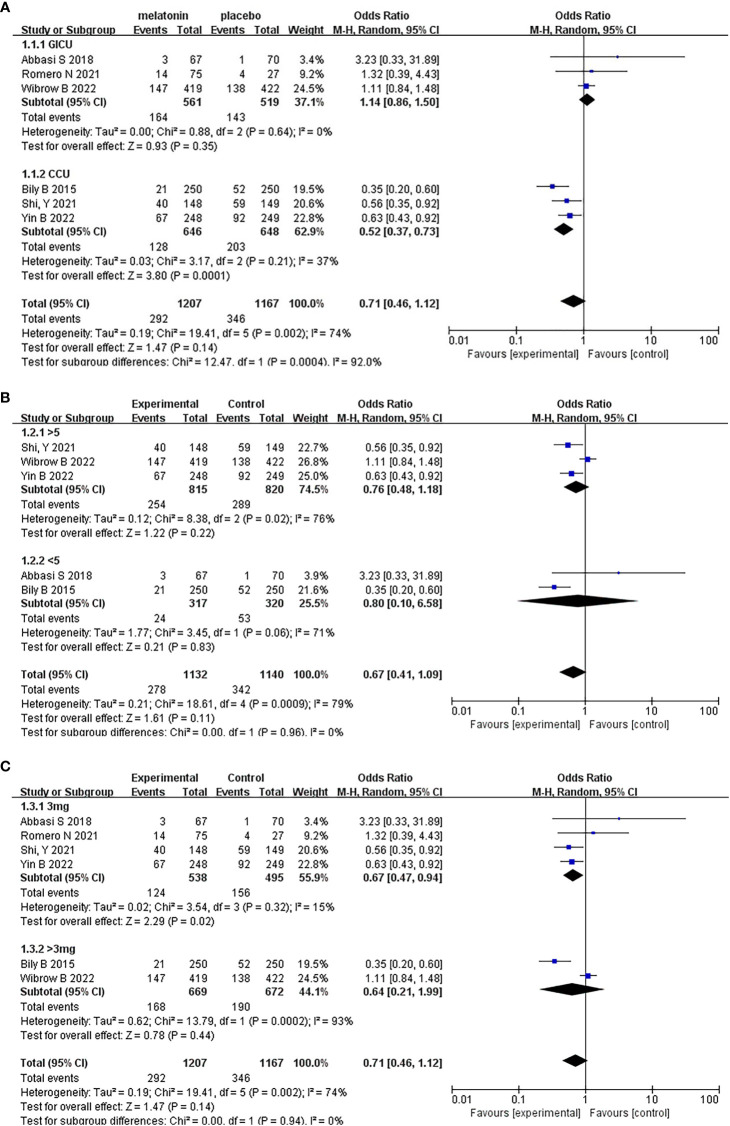
Subgroup analysis of incidence of delirium. **(A)** Type of ICU, **(B)** Dosing duration, **(C)** Daily dosage.

According to the type of ICU, the six studies were divided into two groups for meta-analysis. The results were shown in **Figure 5A**. Based on the above subgroup analysis, the heterogeneity between the two groups was extremely high, reaching a high degree of heterogeneity, which means that inconsistency in ICU type largely influences the results of the meta-analysis. In GICU group, after heterogeneity test, I^2^ = 0%<50% and P=0.64>0.1 of Q test showed there was no heterogeneity in the group. Meta-analysis of three articles showed that melatonin could not reduce the incidence of delirium in GICU patients (Random-effects model: OR, 1.14; 95% CI, 0.86 to 1.50; p=0.35; I^2^ = 0%). Secondly, in CCU group, after heterogeneity test, I^2^ = 37%<50%, and P=0.21>0.1 of Q test, there was no heterogeneity in the group. Meta-analysis of three articles showed that melatonin could reduce the incidence of delirium in CCU patients (Random-effects model: OR, 0.52; 95% CI, 0.37 to 0.73; p=0.0001; I^2^ = 37%). Based on all of the above analyses, melatonin reduced the incidence of delirium in CCU patients, but did not significantly reduce the incidence of delirium in GICU patients.

Based on whether the dosing duration exceeded 5 days, the five studies were divided into two groups for meta-analysis. It should be noted that Romero N (2021) was a single center, retrospective, and objective cohort study. The article did not mention the specific duration, so it cannot be included in the subgroup analysis grouped. The results were shown in **Figure 5B**. Based on the above subgroup analysis, the heterogeneity in the two groups and between the two groups were both high. Moreover, the results of the test for subgroup differences were not statistically significant (Random effects model: OR, 0.67; 95% CI, 0.41 to 1.09; p=0.96; I^2^ = 0%), which means that the duration of administration did not affect the results of the meta-analysis.

On the other hand, based on whether the daily dosage exceeded 3mg, we divided six studies into two groups, the results were shown in **Figure 5C**. Based on the above subgroup analysis, the results of the test for subgroup differences were not statistically significant (Random effects model: OR, 0.71; 95% CI, 0.46 to 1.12; p=0.94; I^2^ = 0%), Which means that different daily dosage did not affect the results of the meta-analysis.

### 4.3 Bias tests

**Figure 6:**
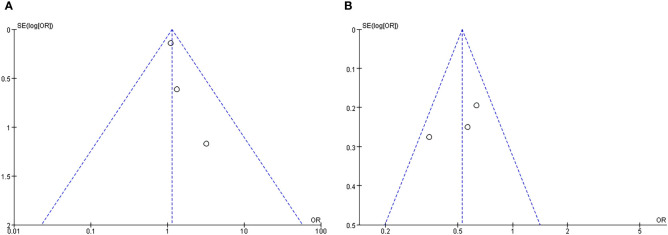
Funnel plot of subgroup analysis about incidence of delirium: **(A)** GICU group; **(B)** CCU group.

Bias tests were conducted according to subgroups and funnel plots were drawn. The results were shown in **Figure 6**. It could be clearly seen that the funnel charts were basically symmetrical, and the bias test showed that all P values were greater than 0.05, so it could be judged that there was no publication bias of this study.

## 5 28-day/30-day all-cause mortality

**Figure 7:**
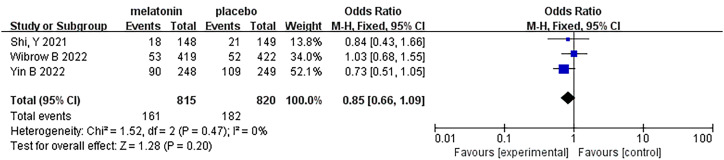
Forest plot and meta-analysis of 28-day/30-day all-cause mortality.

Three articles were analyzed regarding 28-day/30-day all-cause mortality. A total of 1635 patients were included in the studies, of whom 815 received melatonin treatment and 820 received placebo treatment (**Figure 7**). All-cause mortality was similar between the two groups, and no heterogeneity was observed (Fixed-effects model: OR, 0.85; 95% CI, 0.66 to 1.09; p=0.20; I^2^ = 0%).

## 6 Length of ICU stay

**Figure 8:**
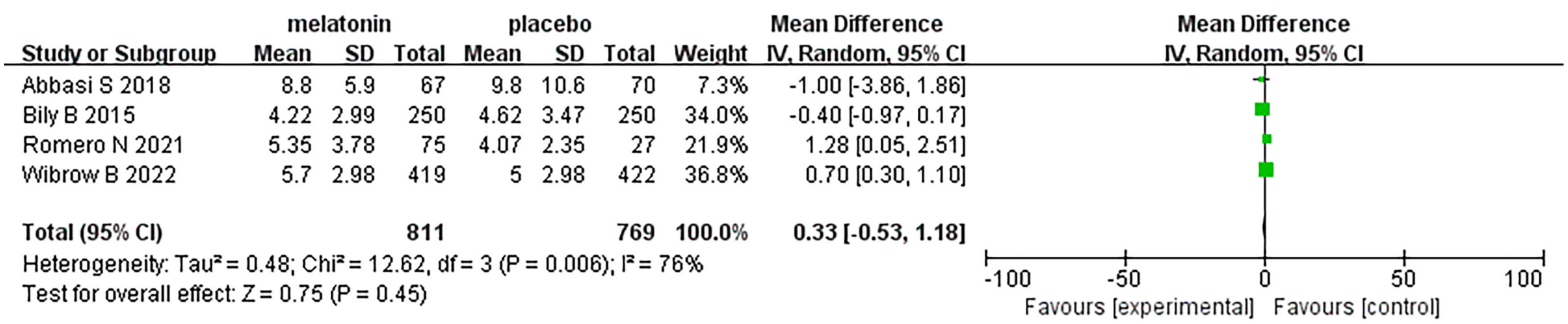
Forest plot and meta-analysis of length of ICU stay.

Four articles were analyzed with respect to length of ICU stay. A total of 1580 patients were included in the studies, of whom 811 received melatonin treatment and 769 received placebo treatment. Because the heterogeneity was considerable (I^2^ = 76%), we used a random-effects model (**Figure 8**). Length of ICU stay was similar between the two groups (Random-effects model: MD, 0.33; 95% CI, -0.53 to 1.18; p=0.45; I^2^ = 76%).

## 7 Length of hospital stay

**Figure 9:**
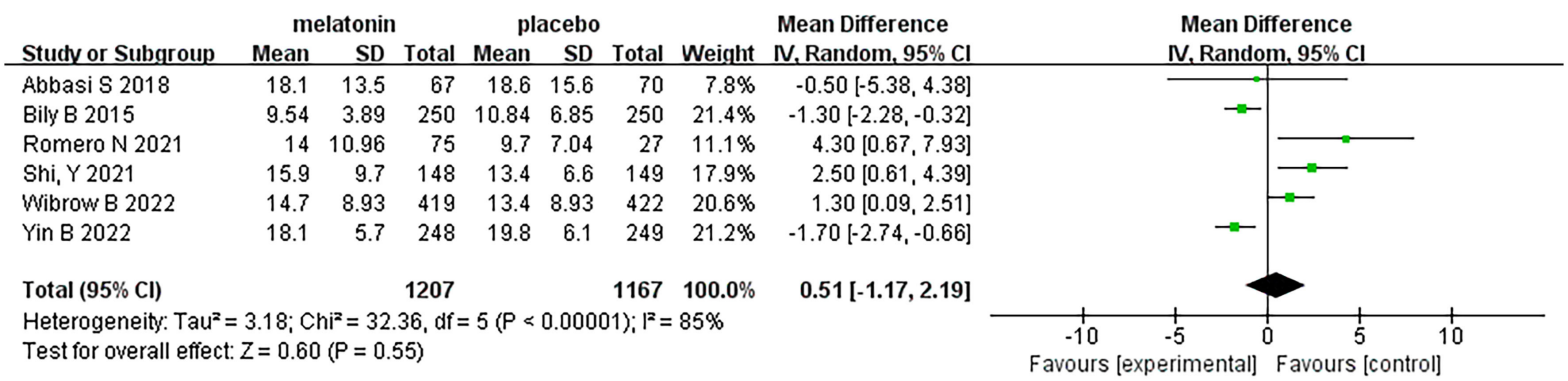
Forest plot and meta-analysis of length of hospital stay.

Six articles were analyzed with respect to length of ICU stay. A total of 2374 patients were included in the studies, of whom 1207 received melatonin treatment and 1167 received placebo treatment. Because the heterogeneity was considerable (I^2^ = 85%), we used a random-effects model (**Figure 9**). Length of hospital stay was similar between the two groups (Random-effects model: MD, 0.51; 95% CI, -1.17 to 2.19; p=0.55; I^2^ = 85%)

## 8 Discussion

Critical illness has been considered a risk factor for the development of delirium, which is associated with increased mortality, prolonged ICU stay, and long-term cognitive decline and poor functional recovery in patients (36, 37). Therefore, any potential benefit of melatonin may be more pronounced in critically ill patients (6, 15, 38). Melatonin is an endogenous hormone secreted by the pineal gland that regulates circadian rhythms (39), and current studies *in vitro* and in animal models have demonstrated the potential anti-inflammatory and antioxidant characteristics of melatonin, suggesting that its use in critically ill patients may have multiple benefits (40, 41).

Our findings suggest that melatonin did not observe a significant advantage in preventing delirium in critically ill patients, but there was a high degree of heterogeneity between studies. In particular, the advantage of melatonin in preventing delirium was not significant in GICU, but in CCU, melatonin was shown to reduce the incidence of delirium, so based on the current findings, it cannot yet be assumed that melatonin is effective in preventing delirium in ICU patients. Although differences in disease severity may attenuate the beneficial effects of melatonin, current treatment options for delirium supported by valid evidence-based medical evidence are limited to preventive strategies with nonpharmacological interventions, such as early activity, light and noise reduction, sleep management, and involving relatives when appropriate (29, 42–44). The recent multicenter randomized controlled trial conducted by De Jonghe A in the Nether-lands for elderly patients with hip fracture also proved that melatonin treatment did not reduce the incidence of delirium (23). This is also consistent with our findings. There are still few studies on melatonin prevention of delirium in critically ill patients, and larger, multicenter randomized controlled studies are needed to support these findings, so the results of this study should be treated with caution.

There may be many reasons for the differences in the effects of melatonin due to different types of ICU, and the specific molecular mechanism of melatonin’s preventive effect on delirium in CCU patients may be closely related to its improvement of vascular endothelial cell function, anti-vasospasm, anti-neuronal apoptosis, improvement of cerebral perfusion, and reduction of microthrombosis (45). Current relevant basic studies have shown that the non-selective opening of the mitochondrial permeability transition pore in mitochondria and the overproduction of reactive oxygen species are important factors promoting heart disease and cardiac dysfunction, and melatonin can improve isoprenaline-hydrochloride-induced heart failure related to mitochondrial dysfunction (46). In addition, Wang J et al. showed that renal ischemia-reperfusion injury can attenuate cardiac diastolic function by increasing cardiomyocyte death and enhancing the inflammatory response, while also disrupting cardiomyocyte energy metabolism, causing calcium overload, and impairing mitochondrial function. In contrast, melatonin reduces cytoplasmic and mitochondrial calcium overload, while melatonin preconditioning attenuates cardiac damage mediated by renal ischemia-reperfusion injury by maintaining myocardial diastolic function and reducing myocardial cell death (47). On the other hand, in GICU, the condition of critically ill patients is relatively complex and the disease types are diverse, and the relatively complex sedative and analgesic regimens used in this group of patients as well as the use of some antipsychotics may alter neurotransmitter levels and subsequently cause direct damage to brain-related functions (48), while it is unclear whether the mixture of these drugs may affect the effectiveness of melatonin.

We completed this meta-analysis under the strict guidance of PRISMA (49), but our analysis still has some limitations. First, the different types of delirium (hyperactive, hypoactive, or mixed delirium) were not distinguished in the study. Second, the CAM-ICU score is only a screening tool and is not the gold standard for delirium diagnosis, and scores are performed by ICU nurses at different levels and may be under-estimated in some studies. Third, melatonin is not regulated in some countries, and therefore the quality of the product may vary depending on the manufacturer, which may lead to differences in the absorption of melatonin in different studies. Fourth, in some studies the authors did not directly provide means and standard deviations for continuous data, so estimated means and standard deviations were used in the analysis of relevant data. Nevertheless, our meta-analysis still had a high level of evidence, the majority of the included studies were randomized controlled trials, and most of the included studies were published in the last five years with comprehensive records of outcome indicators, which significantly increased the credibility of our results.

## 9 Conclusion

Our study of published trials indicates that melatonin reduced the incidence of delirium in CCU patients, but did not significantly reduce the incidence of delirium in GICU patients. The role of melatonin in reducing the incidence of delirium in ICU patients requires further study.

## Data availability statement

The original contributions presented in the study are included in the article/supplementary material. Further inquiries can be directed to the corresponding author.

## Author contributions

Conceptualization, YY, YD, XW and JS; methodology, YY and YD; software, YD and WZ; validation, LW, GW and WZ; formal analysis, XW and YY; investigation, GW, JY and QB; resources, WZ; data curation, YY and YD; writing—original draft preparation, YD and YY; writing—review and editing, LW, XW and JS; visualization, JY and QB; supervision, XW and JS; project administration, XW; funding acquisition, JS. All authors have read and agreed to the published version of the manuscript.
